# Sex differences in the context dependency of episodic memory

**DOI:** 10.3389/fnbeh.2024.1349053

**Published:** 2024-03-01

**Authors:** Aliza A. Le, Linda C. Palmer, Jasmine Chavez, Christine M. Gall, Gary Lynch

**Affiliations:** ^1^Department of Anatomy and Neurobiology, University of California, Irvine, Irvine, CA, United States; ^2^Department of Neurobiology and Behavior, University of California, Irvine, Irvine, CA, United States; ^3^Department of Psychiatry and Human Behavior, University of California, Irvine, Irvine, CA, United States

**Keywords:** episodic memory, unsupervised learning, context, mouse, behavior, sex differences, female, object recognition

## Abstract

Context contributes to multiple aspects of human episodic memory including segmentation and retrieval. The present studies tested if, in adult male and female mice, context influences the encoding of odors encountered in a single unsupervised sampling session of the type used for the routine acquisition of episodic memories. The three paradigms used differed in complexity (single vs. multiple odor cues) and period from sampling to testing. Results show that males consistently encode odors in a context-dependent manner: the mice discriminated novel from previously sampled cues when tested in the chamber of initial cue sampling but not in a distinct yet familiar chamber. This was independent of the interval between cue encounters or the latency from initial sampling to testing. In contrast, female mice acquired both single cues and the elements of multi-cue episodes, but recall of that information was dependent upon the surrounding context only when the cues were presented serially. These results extend the list of episodic memory features expressed by rodents and also introduce a striking and unexpected sex difference in context effects.

## 1 Introduction

Human episodic memory involves the encoding of multiple events into narrative sequences, minimally including the identity and location of items and the order in which they appeared (i.e., ‘what’, ‘where’, and ‘when’ information) (Tulving, 1972, 1983; Staniloiu et al., 2020). The requisite encoding occurs routinely as part of daily life without repetition or explicit rewards, such as a first time walk across a park (Dede et al., 2016); these features distinguish episodic memory from trial and error learning. Despite the rapid and spontaneous nature of such “unsupervised” learning (Barlow, 1989), episodic memories can incorporate a remarkable amount of information and accommodate very different intervals (seconds to minutes) between cues or events (Dede et al., 2016). The incidental, and generally single-trial, nature of episodic encoding presents difficulties for rodent studies but the strong tendency of the animals to investigate novel stimuli or locations can be used to partially compensate for the absence of behavioral shaping (Dix and Aggleton, 1999). Single trials and novelty form the basis for widely used rodent memory tests including Object Location and Novel Object Recognition paradigms (Ennaceur and Delacour, 1988; Ennaceur et al., 1997). Several studies of this type have provided evidence that rats and mice readily learn the identities, locations, and serial order for multiple cues (Hannesson et al., 2004; Kart-Teke et al., 2006; Fouquet et al., 2010; Allen et al., 2014; Barker and Warburton, 2020) under conditions not unlike those found in human studies of episodic memory. However, the extent to which rodents express other characteristics of an episode is uncertain.

We developed a set of relatively simple protocols using multiple odor cues to assess facets of episodic encoding in rodents; this included a task with serial cue presentation to reflect the typical distribution of elements and events encountered across time within a behavioral episode (Staniloiu et al., 2020). Using these paradigms, we verified that mice and rats acquired ‘what’, ‘where’, and ‘when’ information after one-time sampling of the stimulus set (Wang et al., 2018b; Cox et al., 2019; Amani et al., 2021; Le et al., 2022a). Encoding exhibited the temporal flexibility described for human studies in that retention scores were not detectably different for intervals of 30 s vs. 5 min between cues. A subsequent experiment using the same testing procedures provided evidence that transfer of episodic information into long-term storage by rats is promoted by a strong stimulus occurring shortly after the sampling of multiple cues (Quintanilla et al., 2021), an effect that may be analogous to ‘flashbulb memory’ described for humans (Brown and Kulik, 1977; Talarico et al., 2019). Finally, and as with human episodic memory, the hippocampus proved to be critical for learning the three basic elements of an episode in the multiple odor paradigms (Cox et al., 2019).

The present studies tested if another essential feature of human episodic memory – context-dependency (Barak et al., 2013; Staudigl and Hanslmayr, 2013) – is also evident in mice using the above odor-based paradigms and measures of ‘what’ encoding. Retrieval often begins with a memory search for the situation in which the series of events occurred, followed by readout of specific items (Eacott et al., 2005). There is also evidence that the critical process of segmenting the flow of experience into individual episodes depends on a shift in context, as exemplified by the ‘through the doorway’ effect (Smith and Mizumori, 2006; Horner et al., 2016). Studies in rodents have identified circumstances in which incidental encoding of cue pairs is linked with context (Dix and Aggleton, 1999; Eacott and Norman, 2004; Norman and Eacott, 2005; O'Brien et al., 2006; Piterkin et al., 2008; Davis et al., 2013; Seel et al., 2018; Barker and Warburton, 2020). Here we tested if memory retrieval for material presented in sessions with the above noted episodic characteristics is dependent upon the context of initial cue exposure, if such effects differ between the sexes and, for females, between estrous states. There is a sizeable literature describing relative advantages for men and women on different aspects of episodic memory (Herlitz et al., 1999; Loprinzi and Frith, 2018; Asperholm et al., 2019; Voyer et al., 2021) but sex differences in context dependency are rarely considered. Our results indicate that there are marked sex differences in reliance upon context for accessing episodic content and that these context effects are stronger in male than female mice independent of estrous state.

## 2 Materials and methods

Adult male and female sighted-FVB129 wild-type mice (2–5 months old) were used. Animals were group housed (3-5/cage) in rooms (68°F, 55% humidity) on a 12-h light/dark cycle with lights on at 6:30 AM and food and water were given *ad libitum*. Behavioral experiments were performed between 10 AM-3 PM. Mice were not handled prior to experimental procedures. For females, estrous cycle stage was assessed by vaginal lavage (McLean et al., 2012) prior to experimental use to distinguish mice in proestrus [the phase of relatively high circulating and hippocampal estrogen levels (Kato et al., 2013)] from those outside proestrus (i.e., in estrus, diestrus and metestrus). Experiments using the simultaneous 4-odor task evaluated females both within and outside proestrus; other tasks employed females outside proestrus. All experiments were conducted in accordance with the National Institutes of Health Guide for the Care and Use for Laboratory Animals and protocols approved by the Institutional Animal Care and Use Committee at the University of California, Irvine.

### 2.1 General procedures for all behavioral tasks

Naïve mice were tested for the effect of context on evidence for acquisition of cue identity (a.k.a., ‘what’ information) in three tasks that did not entail rehearsal or reward. This included a single-cue (odor) discrimination task, and tasks involving multiple cues presented serially or simultaneously (referred to here as the serial cue and 4-odor ‘what’ tasks, respectively). These particular paradigms were considered important for identification of potential context effects on encoding the identity of multiple vs. individual cues (4-odor vs. single odor tasks), on long term retention of memory for cue identity (24 h from sampling to testing for the 4-odor task only), and on encoding cues presented in series and, thus, over time (serial odor task). All three tasks used odorants previously established to be of equivalent interest to mice (Wang et al., 2018b; Cox et al., 2019; Le et al., 2022b) (Supplementary Table S1). The odorants were dissolved in mineral oil and 100 μL was pipetted onto filter paper immediately before behavioral testing. For odor presentation, the scented filter paper was placed into either a 5.2-cm diameter, 5-cm tall glass jar with a 15-mm sample port hole in a white metal lid (Wang et al., 2018b; Cox et al., 2019; Le et al., 2022b) or, for a subset of mice in the serial cue ‘what’ task, into a 6-cm diameter, 11.25-cm tall pointed plastic cylinder with a 15-cm sample port on the side. The time spent sampling the odors was measured by offline analysis from video recordings, that were collected from all behavioral sessions, by observers blind to group (jars) or by automated quantification of infrared beam breaks (cylinders). Results obtained with the two odor presentation containers were similar and data were combined.

**Figure 1:**
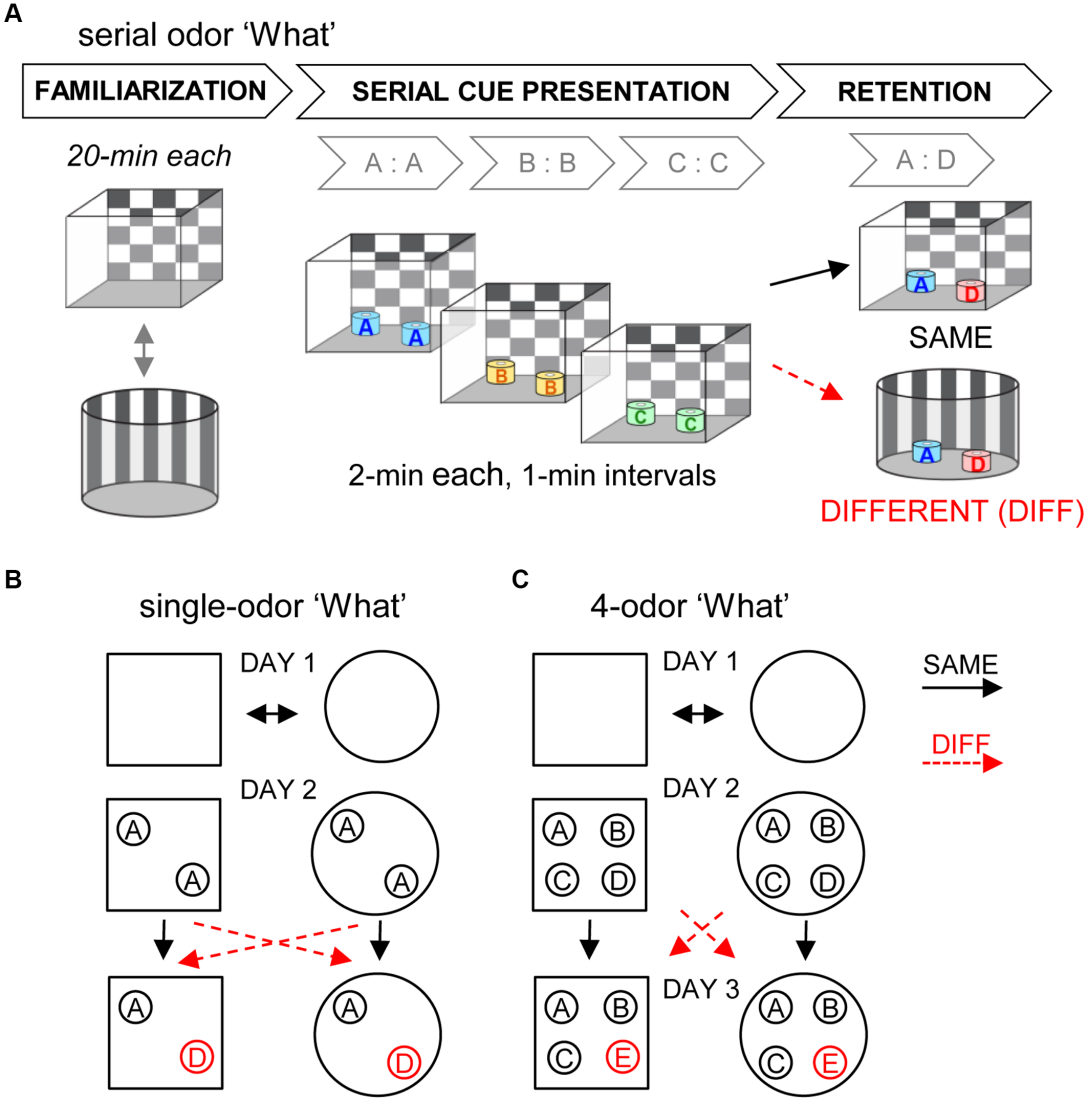
Contextual episodic memory tasks. For all three tasks, mice were familiarized with two distinct chambers for 20-min each on Day 1. **(A)** Serial odor ‘what’ task. On Day 2, mice were presented with a series of 3 odor pairs (2 min each, 1 min between pairs). Two minutes after the last cue exposure, retention was assessed by presentation of an odor pair including previously sampled odor A and a novel odor (e.g., A:D) in either the SAME chamber as initial odor exposures or in the different (DIFF) chamber for 3 min. **(B)** Single-odor task. Mice were exposed to a single odor pair (A:A, circles denote scented jars) for 2 min. After a 10-min delay, they were exposed to odor pair (A:D) in the SAME or DIFF chamber for 3 min. **(C)** 4-odor task. On Day 2 mice were exposed to four odors (A:B:C:D) simultaneously for 5 min. On Day 3, mice were exposed to four odors including 3 familiar and one novel odor (A:B:C:E), and allowed to explore for 5 min.

For all three tasks, on Day 1 mice were first habituated for 20 min each to two distinct arenas (Square: 23w x 30d x14.5 h cm rectangle with a checkerboard pattern on opposing walls, other walls were white. Round: 25-cm diameter cylinder with 24-cm high walls and horizontal stripes on one hemisphere, the other hemisphere was solid gray) (Figure 1A). Distant visual cues were the same across all behavioral sessions. The following day (Day 2), each mouse was placed into one of the two arenas containing unscented containers for a short period of exploration before cue presentation.

### 2.2 Single-odor ‘what’ task

On Day 2 each mouse was placed in one of the two arenas (square or round) containing two odorless containers for 2 min. A pair of identically scented (A:A) jars was then introduced and the mouse was allowed to freely sample the cues for 2 min (Figure 1B) with timing, in this and other tasks, initiated when both odors had been sampled. The cues were removed and the mouse remained in the chamber for 10 min. This holding time was chosen to approximate the total time from initial odor exposure to testing in this and the serial cue ‘what’ task (see below). For testing, the mouse was then placed into either the same arena as initial odor presentation (SAME) or the different but familiar arena (DIFF) that contained jars scented with familiar odor A and novel odor D and were allowed to freely sample the scented jars. Sampling of the test odors during the following 3 min was quantified by observers blind to group from video recordings. For this and other tasks, the arena locations of the novel vs. previously sampled cues were counterbalanced across mice, as was the arena used for the initial odor exposure (i.e., square vs. round). This paradigm is similar to that employed by O'Brien et al. (2006) for analysis of context effects on object recognition in rat with the exception that our studies employed a single trial.

### 2.3 Serial cue ‘what’ task

On Day 2, after 2 min in the unscented arena, each mouse was presented with a sequence of three identical-odor pairs (A:A, B:B, C:C), placed in the same fixed locations in the arena for each pair (Figure 1A). They were allowed to explore each odorant pair for 2 min. There was a 1-min delay between presentations of successive odor pairs. After the last odor pair presentation, the mouse was moved briefly to a holding bin (~2 min). They were then placed into either the SAME arena as initial odor series presentation or the DIFF arena and allowed to explore for 1 min before being presented with a final test odor pair that included one odor from the initial sampling series and one novel odor (e.g., A:D). Sampling of the test odors during the following 3 min was quantified from video recordings or records of beam breaks.

### 2.4 Simultaneous 4-odor ‘what’ task

On Day 2 the mice were placed in one of the arenas (square or round) with unscented jars for 5 min. After a 1 minute delay, four jars, each containing one of 4 distinct scents (A:B:C:D), were placed at four equidistant locations in the field and mice were allowed to investigate the odors for 5 minutes after which they were returned to their home cage (Figure 1C). On Day 3, 24 h after initial odor exposure, the mice were placed into either the SAME or the DIFF arena containing three of the originally sampled cues and one novel cue (A:B:C:E) placed in the original cue locations and were allowed to freely sample the cues for 5 min. With this design the acquisition and retention phases are separated 24-h thereby allowing tests of females that were in proestrus or non-proestrus stages on Day 2 only (i.e., during initial cue sampling).

#### 2.4.1 Statistics

All data presented in the text and figures are group mean ± SEM values. Graphs present either the cue sampling time (in seconds), the discrimination index (DI), or z-scores. The DI for the single-odor and serial cue tasks was calculated as: (t_novel_ – t_familiar_) / (t_total_) x 100, with ‘t’ denoting the sampling time in seconds. For the 4-odor ‘what’ task, the DI was calculated as (t_novel_ – t_mean familiars_) / (t_total_) x 100. Individual z-scores of DIs and total cue sampling times for DIFF relative to SAME group mice were calculated as: (individual value_(DIFF)_ – mean value_(SAME)_)/(standard deviation_(SAME)_). Statistical significance (*p* < 0.05) was determined using GraphPad Prism (v6.0). The two-tailed paired or unpaired *t*-test was used for comparing two groups. In plots of quantitative results, asterisks denote level of significance with ^*^p < 0.05, ^**^p ≤ 0.01, ^***^p ≤ 0.001 and ^****^p ≤ 0.0001.

## 3 Results

### 3.1 Context potently affects retention scores for odor cues in male mice

We tested if encoding the ‘what’ aspect of episodic-like memory is dependent upon context using three different tasks that employed overlapping sets of odor cues and did not entail repeated trials or rewards (Cox et al., 2019). For each, the mice were allowed to initially explore the cues in one of two familiar test chambers (square or round) and then, for retention testing, they were presented with an initially sampled cue (or cues) and a novel cue in either the same chamber as initial sampling (SAME) or the different (DIFF) chamber (Figure 1). In each case, preferential exploration of the novel cue was interpreted as evidence for encoding.

**Figure 2:**
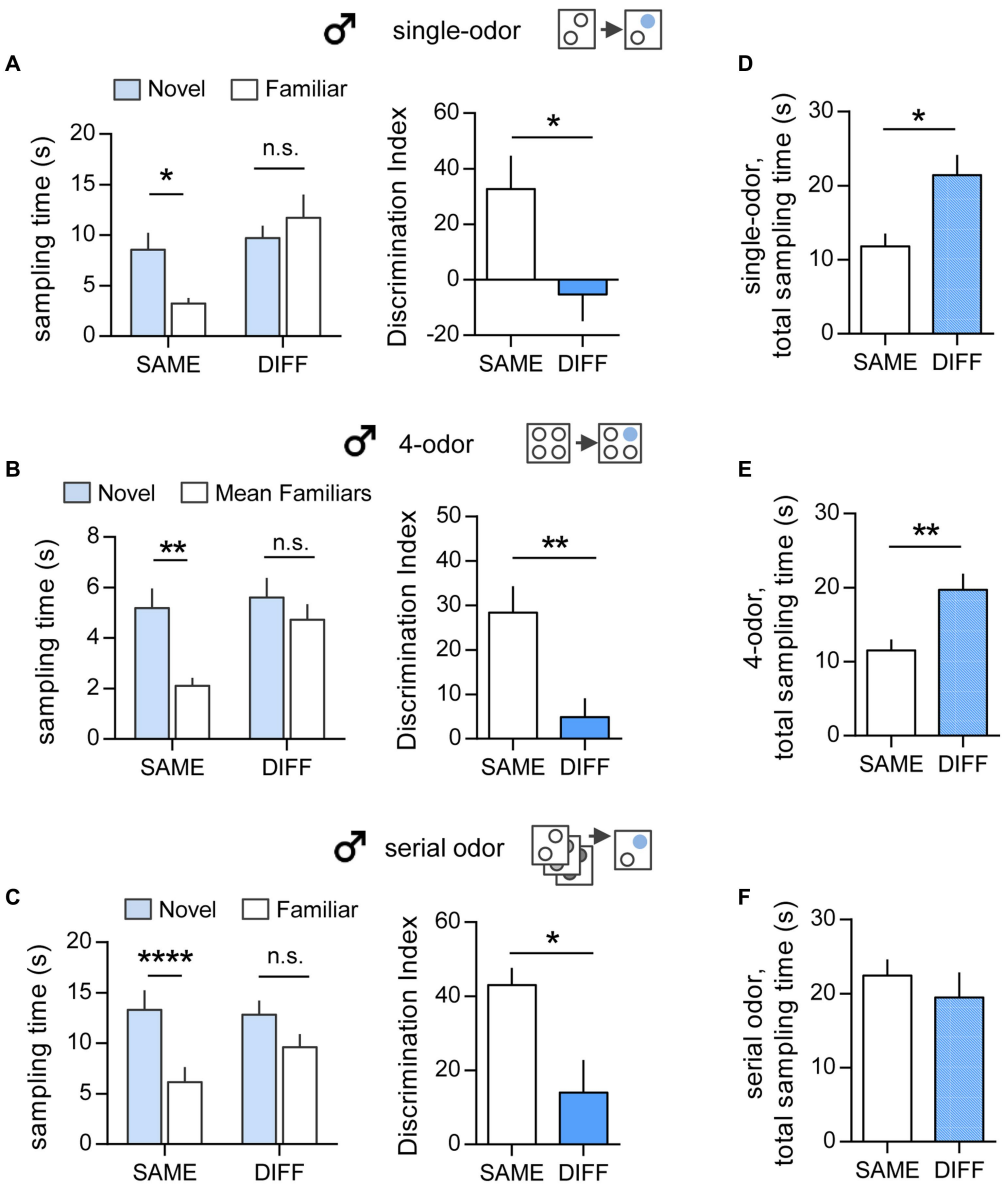
Context influences the performance of male mice in all three ‘what’ tasks. **(A)** Single-odor task. *Left:* Male mice retention-tested in the SAME chamber as initial odor exposure preferentially sampled the novel odor compared to the previously sampled odor (t_(4)_ = 3.01; ^*^p = 0.04; n = 5/group; two-tailed paired t-test), whereas mice tested in the DIFF chamber sampled both odors similarly (t_(7)_ = 0.8218; *p* = 0.438; *n* = 8/group). *Right:* The Discrimination Index (DI) was greater for mice tested in the SAME vs. the DIFF chamber (t_(12)_ = 2.51; ^*^p = 0.027; *n* ≥ 6/group). **(B)** Simultaneous 4-odor task. *Left:* Sampling time was greater for the novel odor vs. mean of times sampling the three previously sampled odors with retention testing in the SAME chamber only (SAME: t_(7)_ = 4.612; ^**^p = 0.002; *n* = 8; DIFF: t_(7)_ = 1.087; *p* = 0.313; *n* = 8). *Right.* The DI was greater for groups tested in the SAME vs. the DIFF chamber (t_(14)_ = 3.22; ^**^p = 0.006; *n* = 8/group). **(C)** Serial odor task. *Left:* Sampling time for novel vs. the previously sampled odor was greater in the SAME as compared to the DIFF chamber (SAME: t_(10)_ = 11.05; ^****^p < 0.0001; *n* = 11; DIFF: t_(16)_ = 2.019; *p* = 0.061; *n* = 17). *Right:* The DI was greater for SAME vs. DIFF group mice (t_(7)_ = 0.8218; *p = 0.02; *n* ≥ 11/group). **(D–F)** The total time sampling the cues during the retention trial was greater in the DIFF chamber compared to the SAME chamber for both the single-odor task (**D**; t_(11)_ = 2.575; *p = 0.026; *n* ≥ 5/group) and the 4-odor task (**E**; t_(14)_ = 3.11; ^**^p = 0.0076; *n* = 8/group). In the serial odor task **(F)**, sampling times were comparable for SAME and DIFF group mice (**F**, t_(26)_ = 0.773; *p* = 0.447; *n* ≥ 11/group). Statistics: Left panels of **A–C**: 2-tailed paired t-test. Right panels of **A–C** and Panels **D–F**: 2-tailed unpaired t-test.

In the simple, single-odor test, male mice spent more time exploring the novel odor vs. the familiar odor when tested in the SAME chamber as initial sampling (Novel 8.57 ± 1.67 vs. Familiar 3.24 ± 0.55 s; *p* = 0.04; two-tailed paired *t*-test). In contrast, mice tested in the DIFF chamber did not show preference for either cue (Novel 9.72 ± 1.21 vs. Familiar 11.7 ± 2.29 s; *p* = 0.44) (Figure 2A, left). This resulted in significantly different DIs for tests in the SAME vs. DIFF arenas (32.8 ± 11.9 vs. -5.26 ± 9.62, respectively, *p* = 0.027, two-tailed unpaired t-test) (Figure 2A, right).

Effects of context were also evident in the long delay, simultaneous 4-odor ‘what’ task (Figure 2B). As previously reported (Cox et al., 2019; Quintanilla et al., 2021), male mice tested 24 h after initial sampling of four odors preferentially explored the novel cue vs. the mean of three familiar cues (*p* = 0.002, two-tailed paired t-test) when retention sessions were conducted in SAME arena. However, when tested in the DIFF context, mice did not exhibit any bias for the novel odor (*p* = 0.313, Figure 2B, left), and, in agreement with this, their DI was notably smaller than those tested in the SAME chamber (DIFF vs. SAME: 4.93 ± 4.21 vs. 28.4 ± 5.93; *p* = 0.006, two-tailed unpaired t-test) (Figure 2B, right).

Finally, and in accord with previous studies using unsupervised learning (Hannesson et al., 2004; Dere et al., 2005; Babb and Crystal, 2006; Cox et al., 2019), male mice presented with a series of cues in the Serial ‘what’ task displayed a clear preference for sampling the novel cue when the retention trial was administered in the SAME chamber (*p* < 0.0001). In contrast, mice tested in the DIFF chamber did not preferentially explore the novel cue (*p* = 0.061, two-tailed paired t-test) (Figure 2C, left). Thus, mice tested in the SAME chamber exhibited a significantly higher DI than those tested in the DIFF chamber (p = 0.02, two-tailed unpaired t-test) (Figure 2C, right).

In male mice there was also an effect of context on the total cue sampling time during the retention trial. In the single-odor paradigm, the total cue sampling time was greater in mice tested in the DIFF chamber than those tested in the SAME chamber (*p* = 0.026, two-tailed unpaired t-test) (Figure 2D). Similarly, in the 4-odor task, the total sampling time was greater for DIFF vs. SAME group mice (*p* = 0.008) despite the 24-h interval between sampling and testing (Figure 2E). In contrast, there was no significant context effect on total sampling time in the serial cue task (*p* = 0.447) (Figure 2F).

### 3.2 Context had little effect on episodic memory in females

**Figure 3:**
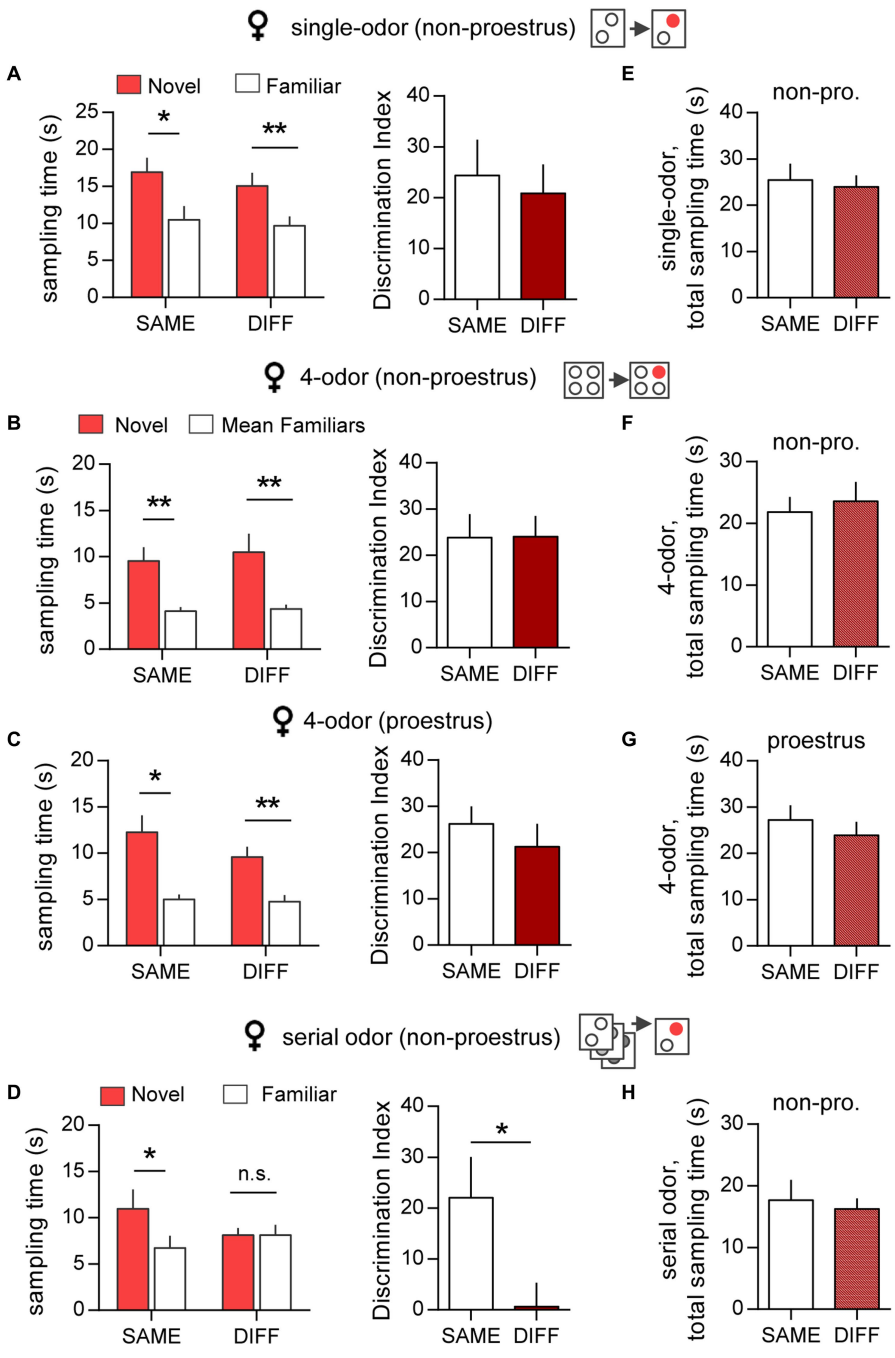
Context has little effect on episodic ‘what’ memory in female mice. **(A)** Single-odor task. *Left:* In females (non-proestrus), the sampling time for the novel odor was markedly greater than the previously sampled odor when tested in either the SAME or the DIFF chamber (SAME: t_(10)_ = 3.04; ^*^p = 0.012; *n* = 11/group. DIFF: t_(8)_ = 3.61; ^**^p = 0.0069; *n* = 9/group). *Right:* Mice tested in SAME and DIFF chamber showed robust and comparable discrimination indices (DIs) (t_(18)_ = 0.031; *p* = 0.713; *n* ≥ 9/group). **(B,C)** Simultaneous 4-odor task for non-proestrus (non-pro) **(B)** and proestrus **(C)** females. *Left:* For both estrous stages, the sampling time for the novel odor was greater than the mean time exploring the three previously sampled odors with retention testing in either the SAME or the DIFF chamber (Shown in **B**, left for non-proestrus mice, SAME: t_(6)_ = 4.187, ^**^ *p* = 0.006, n = 7; DIFF: t_(7)_ = 3.542, ^**^p = 0.009, *n* = 7. Shown in **C**, left for proestrus mice: SAME: t_(3)_ = 4.884, ^*^p = 0.016; *n* = 4. DIFF: t_(6)_ = 5.149; ^**^p = 0.004; *n* = 6). Right: The DIs for both non-proestrus (**B**, right) and proestrus (**C**, right) mice were comparable for SAME and DIFF groups (non-proestrus **(B)**: t_(13)_ = 0.031; *p* = 0.976; *n* ≥ 7/group. Proestrus **(C)**: t_(8)_ = 0.713; *p* = 0.496; *n* ≥ 4/group). **(D)** Serial Odor Task. Left: Females preferentially sampled the novel vs. the previously sampled odor with testing in the SAME chamber but not in the DIFF chamber (SAME: t_(5)_ = 3.239; ^*^p = 0.023; *n* = 6, DIFF: t_(5)_ = 0.004; n.s. *p* = 0.997; *n* = 6). Right: The DI was markedly lower with testing in the DIFF vs. the SAME arena (t_(10)_ = 2.304; ^*^p = 0.044; *n* = 6/group). **(E–H)** The total cue sampling time during the retention trial were similar for SAME and DIFF groups in all tasks (**E**: Single odor task, t_(18)_ = 0.623; *p* = 0.541; *n* ≥ 9/group. **(F)** 4-odor task – non-proestrus, t_(13)_ = 0.421; *p* = 0.68; *n* ≥ 7/group. **(G)** 4-odor task – proestrus, t_(8)_ = 0.750; *p* = 0.475; *n* ≥ 4/group. **(H)** Serial odor task, t_(10)_ = 0.392; *p* = 0.703; *n* = 6/group). Statistics: Panels **(A–D)**, left: 2-tailed paired *t*-test. Panels **(A–D)**, right and Panels **(E–H)**: 2-tailed unpaired *t*-test.

A number of studies have described sex differences in human (Herlitz et al., 1999; Asperholm et al., 2019; Voyer et al., 2021) and rodent (Le et al., 2022b) episodic memory but it is unclear the extent to which such effects extend to context dependency. The present analysis of context effects on episodic ‘what’ encoding indicates that there are indeed major male/female differences. In the single-odor task, female mice that were outside proestrus (non-proestrus) during initial cue sampling, preferentially investigated the novel cue when retention was assessed in either the SAME (*p* = 0.012, two-tailed paired t-test) or DIFF (*p* = 0.007) arena (Figure 3A, left); there was no difference between the DIs for the two groups (*p* = 0.713; two-tailed unpaired t-test) (Figure 3A, right). Comparable results were obtained in the free exploration, 4-odor ‘what’ test. Females outside proestrus during initial sampling, tested in either the SAME or DIFF chamber, preferentially explored the novel odor (SAME: *p* = 0.006, DIFF: *p* = 0.009, two-tailed paired t-test; Figure 3B, left) and the DIs for these two groups were not significantly different (*p* = 0.976; two-tailed unpaired t-test) (Figure 3B, right).

To identify potential effects of changes in circulating and hippocampal estrogen levels across the estrous cycle (Kato et al., 2013), the 4-odor task was repeated in a cohort of female mice that were in a relatively high estrogen state (proestrus) on the day of initial odor exposure. During this session, the total cue sampling time was similar for mice within and outside proestrus (49.50 ± 4.35 s, N = 10 and 48.07 ± 10.73 s, N = 12, respectively; p = 0.44, 2-tailed unpaired t-test). At retention testing, females that initially sampled cues in proestrus successfully discriminated the novel from the familiar odor when tested in either the SAME or DIFF arena (*p* < 0.016; Figure 3C, left), and there was no group difference between the DIs (*p* = 0.496; Figure 3C, right). Thus, performance was similar in females that initially sampled cues during estrous cycle stages with higher (proestrus) and lower (non-proestrus) estrogen levels.

In contrast to performance in the single- and 4-odor tasks, there was a robust effect of context on female performance in the serial odor task. Non-proestrus females tested in the SAME chamber discriminated the novel cue whereas those tested in the DIFF chamber did not (*p* = 0.023, Figure 3D, left). In line with this, the DI was significantly lower in the DIFF vs. the SAME group (*p* = 0.044, Figure 3D, right).

Finally, and in further contrast to performance in males, there was no difference between females tested in the SAME vs. DIFF arena with regard to total cue sampling time during retention testing for any task or group (non-proestrus females in single-odor (*p* = 0.541), 4-odor (*p* = 0.681) or serial odor (*p* = 0.703) tasks; proestrus females in 4-odor task (*p* = 0.47); Figures 3E–H).

**Figure 4:**
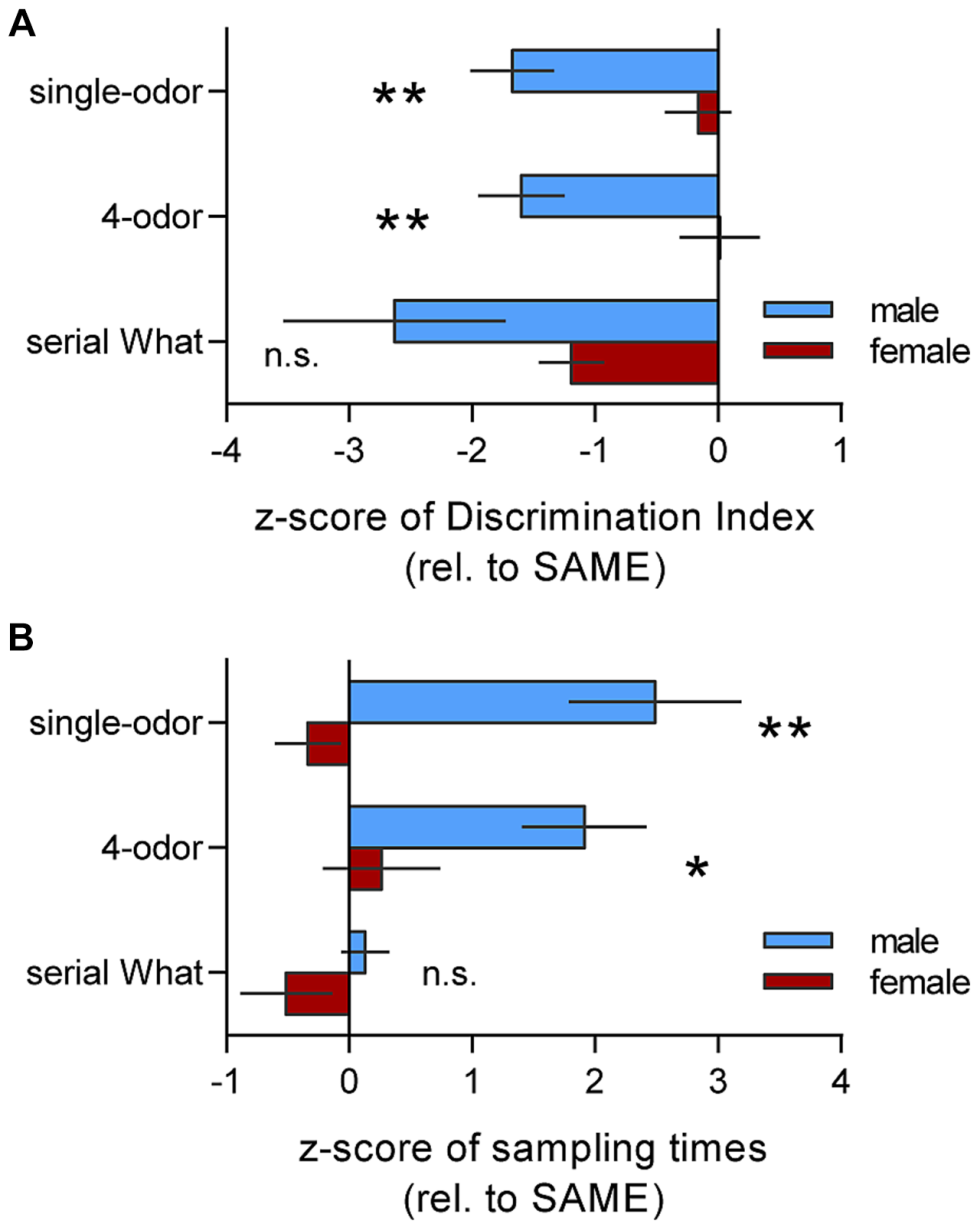
Summary of Z-scores in DIFF group show that context effects on encoding cue identity are sex- and task-specific. Z-scores for mice from the DIFF-group relative to their respective SAME-group were calculated to allow comparison of performance by males and females. **(A)** Z-scores of Discrimination Indices (DIs) show greater effect of context (more greatly negative scores) for males as compared to females for the single-odor and 4-odor tasks (single-odor: t_(14)_ = 3.55; ^**^p = 0.003. 4-odor: t_(14)_ = 2.16; ^**^p = 0.0043; *n* = 8/group). For the serial ‘what’ task, the mean z-scores was more greatly negative in males than females but the difference was not significant (t_(15)_ = 1.146; n.s. *p* = 0.27; *n* ≥ 6/group). **(B)** Plot of Z-scores for total cue sampling times during retention testing shows that for both the single-odor and 4-odor tasks, males spent more time sampling the cues in the DIFF compared to the SAME context whereas females did not (single-odor: t_(14)_ = 3.76; ^**^p = 0.002. 4-odor: t_(14)_ = 2.37; ^*^p = 0.033; male vs. female, n = 8/group). Sex differences were not evident for sampling time Z-scores in the Serial ‘what’ task (t_(16)_ = 1.674; *p* = 0.114; *n* ≥ 7/group). Z-scores were calculated from results presented in Figures 2A–C for males and Figures 3A,B,D for non-proestrus females. Statistics: 2-tailed unpaired *t*-test.

For statistical comparison of context effects on male and female performance, we plotted z-scores for mice tested in the DIFF chamber (normalized to their respective SAME group) for both the discrimination indices and total cue sampling times during retention testing in each of the memory tasks. As shown in Figure 4A, there was a striking effect of context on learning (i.e., the DI) in males but virtually no effect of context in females for both single odor and 4-odor tasks; thus, the male-to-female comparison was highly significant (*p* = 0.003 and *p* = 0.004, single and 4-odor tasks, respectively; two-tailed unpaired t-test). For the serial odor task, both sexes did not discriminate the novel cue when tested in the DIFF chamber and thus the sexes had similar z-scores (*p* = 0.27). Analysis of z-scores for total cue sampling times during retention testing confirmed that the change in context had significantly greater effect on cue exploration in males as compared to females for the single-odor (*p* = 0.002) and 4-odor (*p* = 0.033) tasks (Figure 4B). Results for the serial odor task were again distinctive in that the sexes had similar z-scores for sampling times (*p* = 0.114). Together these results accord with the conclusion that context had greater influence on episodic cue recognition in males than in females.

## 4 Discussion

Context has played an important role in the evolution of thinking about the nature and uses of episodic memory (Federmeier and Sahakyan, 2021) but the manner in which unsupervised experience becomes associated with particular environments or occasions is still poorly understood. Animal studies, with their attendant opportunities for experimental manipulations, could be of importance in addressing this issue but lack an agreed upon description of what constitutes an episodic memory. The paradigms used in the present experiments borrowed key features from a recent episodic memory study in which human participants had a first time walk across a university campus (Dede et al., 2016). Accordingly, the results reported here describe context-dependency in mice using episodic memory paradigms in which multiple cues were sampled on one unsupervised occasion and with intervals between cue encounters varied from seconds (4-odor task) to minutes (serial cue task) in an effort to capture the temporal flexibility that was evident in the human study.

**Figure 5:**
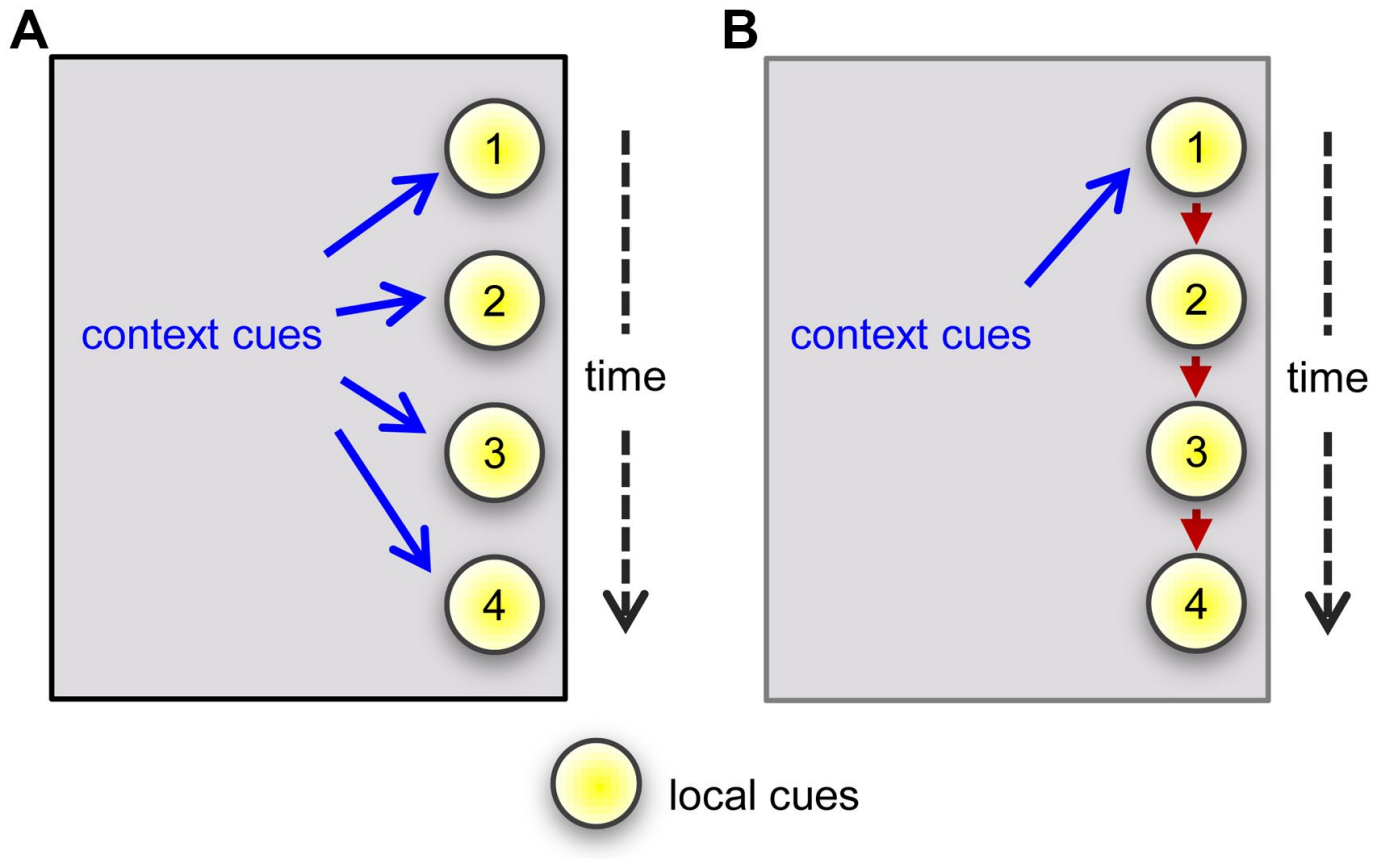
Two hypotheses regarding linkages between environmental context and local cues. The arguments assume that an animal has entered into a previously experienced situation but on this occasion encounters a series of four objects or events that are available for investigation. **(A)** Distant elements in the environment form attachments one by one to each of the items in the sequence. The context will trigger recollection of, and thus enhance familiarity with, the local cues upon re-entry into the environment. The cues will lack context associations in a different environment and hence seem less familiar. **(B)** Linkages form between the context and an item that occurs early in a series and between that early item and a succeeding one. Upon returning to the same environment, the context will prompt a representation of the early cue, and thereby enhance the sense of familiarity (recognition) upon actually encountering that cue. The early cue then prompts the representation of a later one, which strengthens recognition upon its being re-experienced.

The results obtained using three different testing arrangements confirmed that male mice associate context with cues sampled in a single unsupervised episode. Specifically, their normal, robust preference for a novel odor was expressed only when tested in the same environment in which they had previously experienced the comparator odor. The association of cue with context was evident when the mice initially sampled either a single odor or multiple odors, simultaneously or in series, and then were presented with an initially sampled cue(s) vs. a novel one. These results raise the intriguing possibility that the multiple cue cases are a simple extension of the events occurring in the single odor task such that context associates with each of the serially sampled items (Figure 5A). An alternative hypothesis would be that the links form between the multiple cues and the environmental context associates with one of these, which then prompts recall of the others (Figure 5B). Regarding this idea it would be informative to test if the effect of context is stronger for the first cue in a presented series or the first cue investigated by the animal in a single sampling session as in the 4-odor task.

Remarkably, in contrast to effects in males, the testing context did not influence female performance in the single odor or 4-odor tasks: The discrimination indices were comparable in mice tested in either the SAME or the DIFF environment and without obvious effect of the estrous cycle. Males have a significant advantage in spatial learning (Jonasson, 2005; Andreano and Cahill, 2009; Asperholm et al., 2019), suggesting a natural relationship between locations and contexts. That said, the chambers used here were differentiated by shape and wall patterns rather than by landmarks that could be used to specify particular locations, and this design may have influenced responses to the environment. Specifically, it is possible that males directed more attention to the broader environment than females and, accordingly, were more likely to associate it with local cues. Sex differences are described for exploratory and cue sampling behavior (Piber et al., 2018; Chen et al., 2021) and include evidence that males navigate relative to geometric cues in the environment whereas females are influenced by both geometry and landmarks (Korol et al., 2004; Koss and Frick, 2017; Yagi and Galea, 2019). Thus, an alternative possibility is that sex differences in context effects could be due to females having allocated more attention than males to local cues at the expense of encoding information about their surroundings. The first of these hypotheses predicts that recognition strength for the context absent internal cues will be stronger in males than females. The allocation of resources argument predicts that females will outperform males on episodic memory problems other than those that are dependent on space (episodic ‘where’) for which males appear to have clear advantages. Both possibilities are amenable to testing.

Related to the above, males but not females explored the cues for longer periods when tested in the DIFF chamber (where discrimination failed) as compared to the SAME chamber (where discrimination was successful). This observation reinforces the conclusion that the previously sampled local cues were experienced as being novel by the males when encountered in a different context. It has been reported that males continue to explore options in earlier stages of rewarded learning when choices are not clear whereas females tend to select an option and terminate exploration (Chen et al., 2021). In our tasks the males may have continued to sample cues presented in the DIFF chamber because they could not identify the previously sampled cue(s), and thus continued to explore all cues as though they were novel.

The one task in which females did exhibit context dependency entailed presentation of a series of novel cues. “Context” can include local as well as distant cues (Stark et al., 2018). In the single-odor and 4-odor tasks, the retention trial re-introduced familiar cue(s); these may have functioned as landmarks and thus as at least a portion of the “SAME-context” to females regardless of the arena change. Such singular landmarks were not evident in the serial-odor task design, leaving only the chamber as being unchanged through the series. Moreover, this serial presentation may have reinforced the chamber as the constant frame of reference (see Figure 5A), leading to greater influence of this context in the serial “What” task in females.

Strategies in spatial tasks are reportedly influenced by estrous state with proestrus females exhibiting patterns similar to males whereas those outside proestrus exhibit cue-based (allocentric) navigation (Fleischer and Frick, 2023). There is also an extensive literature showing that the performance of female rodents on various learning problems varies with the estrous cycle [(Warren and Juraska, 1997; Frick and Berger-Sweeney, 2001; Luine, 2008; Frick et al., 2015; Lovick and Zangrossi, 2021; Blair et al., 2022; Rocks and Kundakovic, 2023); but also see (Ter Horst et al., 2013; Jayachandran et al., 2022)]. Thus, estrous state might be expected to affect attention to context, and the formation of linkages between elements of context with episodic content (cue identity) in the female groups. However, we did not observe differences in context effects on novelty recognition between proestrus and non-proestrus females, an observation that further discriminates multi-cue episodic learning from more conventional rodent paradigms.

Finally, it is possible that sex differences in brain regions and forms of synaptic plasticity involved in encoding, contribute to sex differences in context effects on episodic memory. Recent studies have shown that in male rodents circuits interconnecting hippocampus with entorhinal, prefrontal and perirhinal cortices are critical for episodic memory (Cox et al., 2019; Allen et al., 2020; Outram et al., 2022) and that hippocampus and its associations with prefrontal and perirhinal cortex are important for linking episodic content with context (Smith and Bulkin, 2014; Barker and Warburton, 2020). Our own chemogenetic studies have shown that, in male mice, acquiring information about cue identity and location relies upon hippocampal afferents from lateral and medial entorhinal cortex, respectively, whereas acquisition of cue-order (episodic ‘when’) is selectively dependent upon hippocampal field CA3 (Cox et al., 2019). The possibility that there are sex differences in the relative importance of regions critical for episodic encoding, or for linking episodic content and context, has not been tested. There is, however, evidence from human imaging studies for differences in regional activation with recall of verbal information (being greater in parahippocampal regions in males, and in dorsolateral prefrontal cortex in females) and with episodic memory performance (being greater in temporal lobe in females) (Loprinzi and Frith, 2018). Similarly, we do not know if forms of synaptic plasticity in the regions linking context with content differ between males and females. Sex differences in threshold and mechanisms of long-term potentiation (LTP), thought to underlie memory encoding, are well-characterized in hippocampal field CA1 (Vierk et al., 2012; Oberlander and Woolley, 2016; Wang et al., 2018a; Le et al., 2022b) and distinguish plasticity in this region from forms of LTP in other systems including the entorhinal afferents to the dentate gyrus (Wang et al., 2018c). It is possible that there are as yet unappreciated sex differences in plasticity within cortical fields that associate context with episodic content giving rise to differences in the strength of these associations.

In summary, a rodent type of episodic memory that bears many similarities to the version described for humans is strikingly dependent on context in males but not females. It is suggested that this is a consequence of sex differences in learning strategies and their possible neuronal substrates.

## Data availability statement

The original contributions presented in the study are included in the article/Supplementary material, further inquiries can be directed to the corresponding authors.

## Ethics statement

The animal study was approved by Institutional Animal Care and Use Committee at the University of California, Irvine. The study was conducted in accordance with the local legislation and institutional requirements.

## Author contributions

AL: Formal analysis, Investigation, Writing – original draft, Writing – review & editing, Conceptualization. LP: Conceptualization, Formal analysis, Investigation, Writing – original draft, Writing – review & editing. JC: Formal analysis, Investigation, Writing – review & editing. CG: Writing – original draft, Writing – review & editing. GL: Conceptualization, Formal analysis, Writing – original draft, Writing – review & editing.
