# Supplementary material for: Sex differences in the context dependency of episodic memory
Source: Front Behav Neurosci. 2024 Mar 1;18:1349053. doi: 10.3389/fnbeh.2024.1349053 (PMC10956361; doi:10.3389/fnbeh.2024.1349053)
Supplement: Supplementary file 1 [file Table_1.DOCX]

**Table 1. List of Odorants used for Behavioral paradigms.**

| **Odorant ID** | **Odorant  (name, *company*)** | **Concentration  (odorant : mineral oil)** |
| --- | --- | --- |
| A | (+) -Limonene (>97% purity, *Sigma- Aldrich*) | 1 : 4000 |
| B | Cyclohexyl ethyl acetate (>97%, *International Flavors & Fragrances Inc.*) | 1.97 : 4000 |
| C | Citronellal 96% (~96%, *Alfa Aesar*) | 1.5 : 4000 |
| D | Octyl aldehyde 99% (~99% *Acros Organics*) | 1.5 : 4000 |

Each odorant was diluted in mineral oil of specific concentrations prior to testing.
